# Cooperative targeting of NF-κB enhances ferroptosis-driven HCC therapy with Alisertib and Donafenib

**DOI:** 10.3389/fcell.2025.1637767

**Published:** 2025-08-13

**Authors:** Qiong Zhou, Rui Wang

**Affiliations:** Laboratory of Medical Oncology, Jinling Hospital, Affiliated Hospital of Medical School, Nanjing University, Nanjing, Jiangsu, China

**Keywords:** hepatocellular carcinoma, Donafenib, Alisertib, ferroptosis, NF-κB signaling pathway

## Abstract

**Background:**

Hepatocellular carcinoma (HCC) is a major cause of cancer-related mortality worldwide. It is often diagnosed at advanced stages, which limits treatment options. Although Donafenib is a standard therapy for advanced HCC, its effectiveness is often reduced by treatment failures. Alisertib, an Aurora-A kinase inhibitor, shows promise in enhancing the cytotoxic effects of Donafenib. This study investigates the combined therapeutic effects of these two agents.

**Methods:**

Synergistic cytotoxicity was assessed via CCK-8 and colony formation assays. Ferroptosis activation was quantified through flow cytometry, lipid peroxidation, and measurements of reactive oxygen species (ROS), intracellular Fe^2+^, and GSH/GSSG. Mechanistic studies involved immunofluorescence for NF-κB/p65 localization, along with Western blotting, qPCR, and dual-luciferase reporter assays to evaluate protein and gene expression. Chromatin immunoprecipitation (ChIP) experiments were performed to analyze the binding of NF-κB/p65 to its endogenous promoters. *In vivo* xenografts were established to evaluate the antitumor efficacy and potential side effects of the combination treatment, supported by histological and immunohistochemical analyses.

**Results:**

Optimal synergistic concentrations (Alisertib 2.5 µM + Donafenib 10 µM for HCCLM3; 5 µM for Huh7) induced profound ferroptotic cascades, evidenced by elevated ROS, lipid peroxides, and Fe^2+^ accumulation concurrent with GSH depletion. The co-treatment potently inhibited p65 nuclear translocation while stabilizing IκBα, thereby suppressing NRF2-mediated antioxidant transcription. Xenograft models demonstrated marked tumor volume reduction with preserved organ architecture and hematological parameters, confirming clinical translatability.

**Conclusion:**

Alisertib is identified as a potent enhancer of Donafenib-induced ferroptosis through inhibition of the NF-κB/NRF2 pathway. This suggests a novel combinatorial strategy that targets ferroptosis through NF-κB inhibition. Further research is needed to translate these promising results into clinical practice.

## Introduction

Hepatocellular carcinoma (HCC) is a leading cause of cancer-related deaths worldwide, primarily due to late-stage diagnoses and treatment challenges (Siegel et al., 2024; Li et al., 2024). Treatment options include surgery, ablation, intra-arterial therapies, radiation, and systemic therapies with targeted and immunotherapies (Vogel et al., 2022). However, many patients present with advanced disease, resulting in poor treatment outcomes. Donafenib, a multi-kinase inhibitor and standard treatment for advanced HCC, significantly improves overall survival in Phase II-III trials while maintaining a favorable safety profile (Qin et al., 2021; Alawyia and Constantinou, 2023). Nonetheless, its clinical benefits are still limited. Therefore, understanding the mechanisms of Donafenib-induced cell death is crucial for exploring combination therapies to enhance its therapeutic impact.

Ferroptosis is a cell death mechanism regulated by iron-dependent lipid peroxidation, making it a promising therapeutic target in oncology, particularly for HCC(Ajoolabady et al., 2023; Liang et al., 2023; Du et al., 2024). Unlike apoptosis and necrosis, ferroptosis represents a balance between oxidation and antioxidation. When this balance is disrupted, excess lipid peroxidation can impair normal cellular functions, ultimately leading to ferroptotic cell death (Tang and Kroemer, 2020; Tang et al., 2024). Recent studies suggest that promoting ferroptosis could represent a novel approach to overcome resistance mechanisms in HCC (Zhao S. et al., 2022; Zhou et al., 2023; Tang et al., 2020). Gao et al. demonstrated that YAP/TAZ transcription factors confer sorafenib resistance in HCC through TEAD-ATF4 axis-mediated SLC7A11 upregulation, which critically suppresses ferroptosis (Gao et al., 2021). Zheng et al. further demonstrated that Donafenib-GSK-J4 combination therapy induces ferroptosis in HCC through HMOX1-mediated iron accumulation, mechanistically linking heme oxygenase activation to lethal lipid peroxidation cascades, thereby revealing druggable vulnerabilities in refractory liver cancer (Zheng C. et al., 2023).

Alisertib, a highly selective Aurora-A kinase inhibitor, exerts its therapeutic effects via high-affinity enzyme binding, triggering mitotic catastrophe through aberrant spindle assembly and chromosomal missegregation (Zhou et al., 1998). Emerging as a multitargeted small-molecule agent, alisertib synergistically enhances conventional chemotherapies and targeted anticancer regimens through concurrent pathway blockade, positioning it as a promising chemosensitizer in precision oncology (Du et al., 2021; Zheng D. et al., 2023). Ongoing early-phase trials (Phase I-II) are systematically evaluating Alisertib monotherapy and its combinatorial potential with radiotherapy, cytotoxic regimens, molecular-targeted agents, and immunomodulators across multiple clinical cohorts (Zhou et al., 2024). Our previous studies demonstrated that Aurora-A expression is crucial for mediating chemotherapy and radiotherapy resistance in HCC. Specifically, we revealed Aurora-A induces chemoresistance by modulating the NF-κB/miR-21/PTEN axis, while concurrently promoting radiotherapy resistance through NF-κB activation and apoptotic gene upregulation (Shen et al., 2019; Zhang et al., 2014). Furthermore, we investigated Aurora-A inhibitors’ capacity to resensitize HCC cells to sorafenib via AKT/MAPK pathway modulation, establishing these inhibitors as promising therapeutic candidates against HCC’s aggressive biology (Zhang et al., 2018b). Nevertheless, whether Aurora-A inhibition enhances Donafenib efficacy remains undetermined. Although preliminary evidence suggests Alisertib may potentiate tumor sensitivity to multiple therapies, its role in ferroptosis induction within HCC requires systematic exploration.

This study investigates the synergistic potential of alisertib in enhancing Donafenib-induced ferroptosis in HCC while deciphering the mechanistic underpinnings. Through systematic evaluation of Alisertib/Donafenib combinatorial treatment in HCC models, we establish optimized therapeutic thresholds (alisertib: 2.5 μM *in vitro*; 30 mg/kg *in vivo*) that augment Donafenib’s ferroptotic efficacy via NF-κB/NRF2 axis suppression. Crucially, subtoxic alisertib co-treatment amplifies Donafenib-driven lipid peroxidation cascades while disrupting NRF2-mediated antioxidant defenses. These mechanistically defined interactions provide a rational basis for developing ferroptosis-focused combination regimens to improve survival outcomes in advanced HCC.

## Materials and methods

### Cell culture

HCC cell lines HCCLM3 and Huh7 were obtained from the Cell Bank at the Chinese Academy of Sciences. Cells were cultured in a humidified incubator at 37°C with 5% CO_2_. The DMEM medium (C11995500BT, Gibco, United States) was supplemented with 10% fetal bovine serum (FSP500, ExCell Biotech, China) and 1% penicillin/streptomycin (C100C5, NCM Biotech, China) for optimal growth.

### Chemicals and reagents

Donafenib (HY-10201S), Alisertib (HY-10971), and Ferrostatin-1 (HY-100579) were acquired from MedChemExpress (MCE) Corporation located in Shanghai, China. Z-VAD-FMK (S7023), 3-MA (3-Methyladenine, S2767), Necrostatin-1 (Nec-1, S8037), Belnacasan (VX-765, S2228), and PMA (S7791) were sourced from Selleck Chemicals in Houston, TX. Dimethyl sulfoxide (DMSO) was supplied by Sigma-Aldrich, also in Shanghai, China.

### Western blotting

Total protein extraction utilized the Protease and Phosphatase Inhibitor Cocktail (P002, NCM Biotech, China). Cytoplasmic and nuclear proteins were isolated using the Nuclear and Cytoplasmic Protein Extraction Kit (KGB5302, KGI Biotechnology) according to the manufacturer’s instructions. Protein concentrations were measured with the BCA assay (ZJ102 kit, Epizyme Biomedical Technology). Samples were mixed with a 5 × loading buffer (LT103, Epizyme Biomedical Technology) and heated at 95°C for 10 min. We loaded 30 µg of total proteins and 60 µg of phosphorylated proteins onto 12.5% SDS-PAGE gels, followed by transfer to 0.2 µm PVDF membranes (Merck, Millipore). Membranes were blocked with 5% skim milk or BSA for 1 h to reduce non-specific binding, then incubated overnight at 4°C with primary antibodies (see Supplementary Table S1). Species-specific secondary antibodies were applied for 1–2 h at room temperature. Finally, protein bands were visualized using Enhanced Chemiluminescent detection (P10060, NCM, China).

### RNA extraction and RT-qPCR

Total RNA was extracted from biological samples using the RNAex Pro Reagent (AG21101, Accurate Biology, China), followed by a purification step in accordance with the protocols outlined in the SteadyPure RNA Extraction Kit (AG21024, Accurate Biology, China). The quality of the isolated RNA was assessed by quantifying both concentration and purity using a NanoDrop One spectrophotometer (Thermo Fisher Scientific, MA, United States). For complementary DNA (cDNA) synthesis, 2 µg of total RNA was reverse transcribed using the Evo M-MLV RT Premix (AG11706, Accurate Biology, China). Subsequently, quantitative real-time PCR (qPCR) analysis was performed with 100 ng of the synthesized cDNA as the template for amplification. mRNA expression levels were evaluated using the SYBR Green Premix Pro Taq HS qPCR Kit (Rox Plus, AG11718, Accurate Biology, China) on a QuantStudio 1 Real-Time PCR System developed by Applied Biosystems, a subsidiary of Thermo Fisher Scientific, MA, United States. Detailed information regarding the primer sequences used in this study is provided in Supplementary Table S2.

### Hematoxylin-eosin (H&E) staining and immunohistochemistry (IHC)

Tumor-bearing mice were euthanized, and tumors and surrounding organs were collected to assess systemic toxicity. Specimens were fixed in 4% paraformaldehyde for at least 24 h, then embedded in paraffin and sectioned to 3–5 μm for H&E staining (BL700A, Biosharp, China). For IHC, sections were deparaffinized, rehydrated, and subjected to antigen retrieval. After blocking to reduce non-specific binding, sections were incubated with primary and secondary antibodies, and nuclei were counterstained with hematoxylin.

### Immunofluorescence

HCC cells were seeded in confocal dishes and allowed to adhere. After 48 h of drug treatment, the medium was discarded. Cells were fixed with 4% paraformaldehyde for 20 min, permeabilized with 0.3% Triton X-100 for 15 min, and blocked with goat serum for 1 h. They were incubated overnight at 4°C with an anti-p65 antibody (1:200, #8242, Cell Signaling Technology, United States), followed by a 1-hour incubation with Alexa Fluor® 488-AffiniPure goat anti-rabbit IgG (Jackson ImmunoResearch Laboratories, United States). Nuclei were stained with DAPI (C1002, Beyotime, China) for 5 min.

### Transmission electron microscopy (TEM)

HCC cells treated with Donafenib and/or Alisertib were fixed overnight at room temperature using a fixative (G1102, Servicebio, China). This was followed by fixation with osmium tetroxide, dehydration, and embedding in resin. Ultrathin sections (60–80 nm) were prepared using an ultramicrotome (Leica EM UC7) and stained with heavy metals (lead and uranium).

### Cell viability and IC_50_ assays

Cell viability was assessed using the CCK8 kit (BS350, Biosharp, China), with HCC cells seeded in 96-well plates and treated with the respective treatments for 0–72 h. Every 24 h, the medium was replaced with CCK8 reagent, and after a 1.5-hour incubation, optical density was measured at 450 nm to evaluate cell viability.

### Cell death and cell death manner assessment

After a 48-hour exposure to pharmacological agents, cells were harvested from 6-well plates and stained with Annexin V and propidium iodide (PI) (KGA1102, KeyGEN, China) for flow cytometry analysis on a Beckman Coulter instrument. Inhibitors used included Z-VAD-FMK (20 µM) for apoptosis, 3-MA (10 µM) for autophagy, Nec-1 (10 µM) for necroptosis, VX-765 (20 µM) for pyroptosis, and Ferrostatin-1 (10 µM) for ferroptosis.

### Drug synergy determination

Based on the established IC_50_ values for Donafenib and Alisertib, a 7 × 7 concentration gradient matrix was formulated. The concentrations for Donafenib were 0, 0.625, 1.25, 2.5, 5, 10, and 20 μM, while the concentration gradient for Alisertib included 0, 1.25, 2.5, 5, 10, 20, and 40 μM. HCCLM3 and Huh7 cells were cultured in 96-well plates according to this matrix design. Subsequently, drug synergy scores were calculated using SynergyFinder software, and a heatmap representing the synergistic interactions was generated using the built-in zero interaction potency (ZIP) approach (Ianevski et al., 2022).

### Clone formation assay

HCCLM3 and Huh7 cells were seeded in six-well plates at a density of 600 and 800 cells per well, respectively, with medium changes every 2–3 days until colonies exceeded 50. After treatment, the colonies were fixed with 4% paraformaldehyde and stained using a 0.1% crystal violet solution.

### 5-Ethynyl-20-deoxyuridine (EdU) assay

The EdU incorporation assay assessed cellular proliferation differences by treating adherent cells in six-well plates with compounds for 48 h, followed by 10 µM EdU for 2 h. Staining was performed per the manufacturer’s instructions (C0078, Beyotime, China), with nuclei counterstained using Hoechst 33,342.

### LPO and cytosolic ROS assay

HCCLM3 and Huh7 cells were cultured in 6-well plates at 4 × 10^5^ cells per well and treated with pharmacological compounds for 48 h. Lipid peroxides (LPO) and intracellular reactive oxygen species (ROS) levels were measured using the C11-BODIPY 581/591 and DCFH-DA probes, respectively, following the manufacturer’s instructions (Elabscience, China).

### Total iron content determination

After 48 h of treatment with pharmacological agents, approximately one million cells from each group were collected and incubated with the iron probe following the manufacturer’s guidelines (E-BC-K880-M, Elabscience, China). Absorbance was measured at 593 nm using a microplate reader to evaluate the results.

### Total glutathione determination

Cells were plated in 6-well culture plates at 4 × 10^5^ cells per well and incubated overnight for adherence before undergoing specified treatments. Following treatment, total intracellular glutathione (GSH/GSSH) was quantified using an assay kit (E-BC-K097-M, Elabscience, China) following the manufacturer’s protocol.

### Malondialdehyde assay (MDA)

The intracellular concentration of malondialdehyde (MDA) was measured using a Colorimetric Assay Kit from Elabscience (E-BC-K028-M, China), with optical density assessed at 532 nm using a microplate reader for precise quantification.

### RNA sequencing

HCCLM3 cells were treated with Donafenib and/or Alisertib for 48 h. Total RNA was extracted using Trizol reagent from Invitrogen Life Technologies, following the manufacturer’s instructions. Sequencing libraries were constructed according to standardized protocols and subsequently subjected to high-throughput sequencing on the Illumina NovaSeq 6,000 platform, operated by Novogene Co., Ltd., China.

### Bioinformatics analysis

Differentially expressed genes (DEGs) were identified from transcriptomic data collected from drug-treated HCC tissues, as well as from adjacent non-tumorous samples, using information sourced from The Cancer Genome Atlas (TCGA). The analytical process was conducted using the R package DESeq2, which is well-regarded for its robustness in comparing gene expression levels across different conditions. Subsequently, we conducted Gene Set Enrichment Analysis (GSEA) (Subramanian et al., 2005) and single-sample Gene Set Enrichment Analysis (ssGSEA) (Hänzelmann et al., 2013) to evaluate the enrichment of specific biological pathways. This analytical approach was instrumental in illuminating the biological significance of our findings, allowing us to gain deeper insights into the underlying mechanisms associated with the differentially expressed genes identified in our study.

### Dual-luciferase reporter assay

HEK293T cells were seeded in 24-well plates at 70%–90% confluency and transfected in Opti-MEM medium with a total of 0.5 μg of NFE2L2 firefly luciferase reporter plasmid, 0.5 μg of RELA overexpression plasmid (or an empty vector control), and 0.02 μg of pRL-TK Renilla luciferase internal control plasmid using Lipofectamine 3000. After 6 h, the medium was replaced with complete DMEM containing 10% FBS. Cells were harvested 24 h post-transfection for luciferase activity measurement using the Dual-Luciferase Reporter Assay System (Promega). Firefly luciferase activity was normalized to Renilla luciferase values to facilitate data analysis.

### Chromatin immunoprecipitation (ChIP) assay

The ChIP assay was performed using the ChIP-IT® Express Enzymatic Kit (Active Motif, United States) with the ChIP-IT® Control Kit—Human to ensure experimental validity. Briefly, cells were crosslinked with 1% formaldehyde, lysed, and enzymatically sheared to generate DNA fragments (200–1,500 bp). Chromatin complexes were immunoprecipitated with anti-NF-κB/p65 antibody or control rabbit IgG, followed by qPCR analysis using specific primers targeting the κB1 (−820) and κB2 (−220) sites: κB1 forward 5′-TGCACTCGGTAATCGGCTACA-3′, reverse 5′-GGGAGCTAACGGAGACCT-3’; κB2 forward 5′-ACTCCCACGTGTCTCCATTC-3′, reverse 5′-CGATTACAGCATGTTGTGGTATT-3’.

### Xenograft mouse tumor model

All animal care and experimental procedures were approved by the Comparative Medicine Center of the Eastern Theater General Hospital, strictly adhering to the International Guiding Principles for Animal Research. The study involved female BALB/c mice, aged 5–6 weeks, sourced from GemPharmatech, China. To establish a tumor model, HCCLM3 cell lines were cultured in the logarithmic growth phase, and approximately 5 × 10^6^ cells were subcutaneously injected into the right anterior axilla of each mouse. Once tumors reached an estimated volume of 100 mm^3^ (calculated using V = ½ ab^2^, where ‘a’ is the longest diameter and ‘b’ is the shortest), treatment began.

### Statistical analysis

Data analysis and visualization were conducted using R software (version 4.2.1) alongside GraphPad Prism 9.0 (version 9.5.1). Numerical results are presented as mean ± standard deviation (SD). Statistical significance was evaluated using t-tests, unpaired t-tests, and one-way analysis of variance (ANOVA), with equal variances confirmed. To enhance reliability, all analyses included biological replicates performed in triplicate. Statistical significance thresholds were set at *P < 0.05; **P < 0.01; ***P < 0.001; and ****P < 0.0001.

## Results

### Alisertib at low dose enhances Donafenib’s cytotoxicity against HCC cells

**FIGURE 1:**
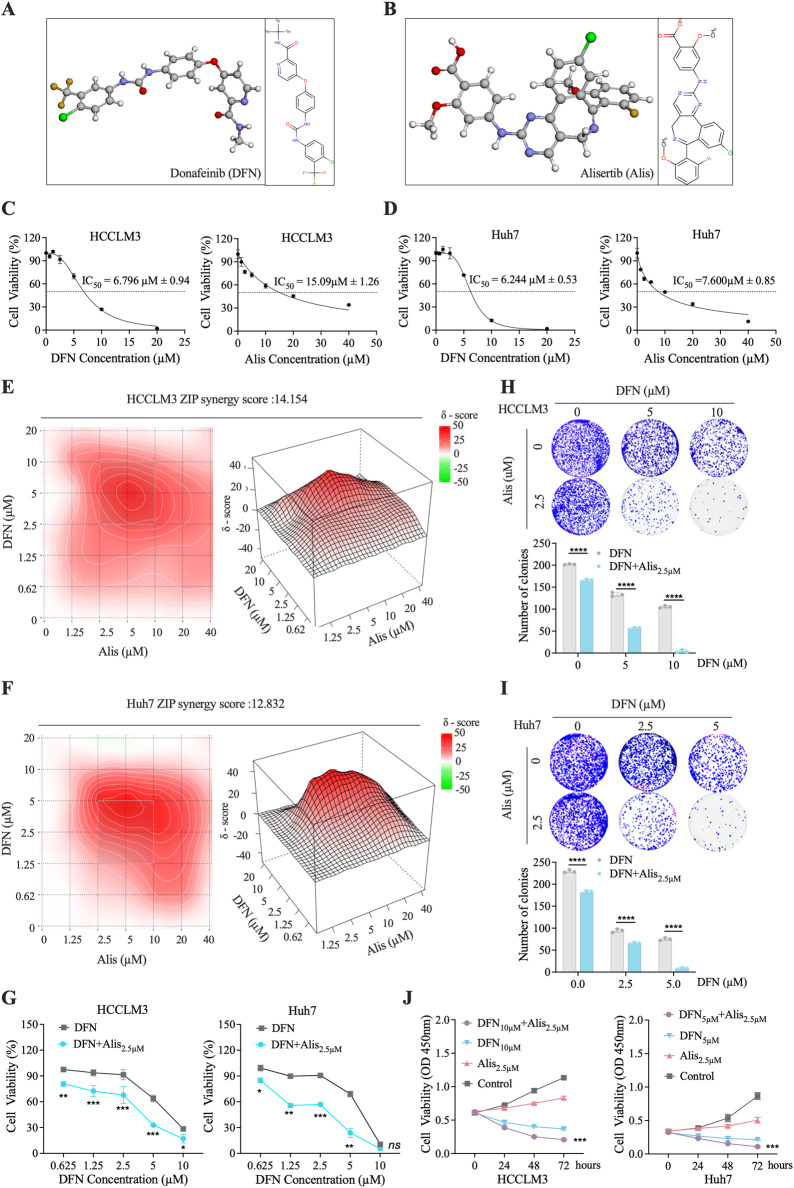
*In Vitro* Assessment of the Synergistic Cytotoxicity of Alisertib and Donafenib in HCC Cells. **(A,B)** The molecular 3D and 2D structures of Donafenib (DFN) and Alisertib (Alis). **(C,D)** A dose-response analysis was conducted to evaluate the effects of Donafenib and Alisertib on HCCLM3 and Huh7 cells. **(E,F)** Heatmaps illustrating the response of HCCLM3 and Huh7 cells to different concentrations of Donafenib and Alisertib. To further identify the optimal dosage of Donafenib, additional experiments assessing cell viability **(G)** and clonogenicity **(H,I)**. **(J)** The relative cell viability of HCCLM3 and Huh7 cells within 72 h. (n = 3, *P < 0.05, **P < 0.01, ***P < 0.001, ****P < 0.0001, ns, non-significant; DFN, Donafenib; Alis, Alisertib).

*In vitro* experiments demonstrated that Alisertib synergistically enhances the cytotoxicity of Donafenib against HCC cells. The molecular structures of Donafenib and Alisertib are shown in Figures 1A,B. Initial biological assessments were performed using the CCK-8 assay on HCCLM3 cells, generating dose-response curves (Figure 1C), with a parallel analysis on the Huh7 cell line (Figure 1D). Both drugs exhibited concentration-dependent anti-proliferative effects, and IC_50_ values were calculated as illustrated in Figures 1C,D. Based IC_50_ values, we established a concentration gradient and cellular viability matrix to compute drug ZIP synergy scores using the online SynergyFinder software (Figures 1E,F). The average synergy scores were 14.15 for HCCLM3 cells and 12.83 for Huh7 cells, with peak synergy regions marked by white dashed boxes achieving scores of 26.09 and 30.37, respectively. ZIP scores above 10 indicate a strong synergistic effect in inhibiting tumor proliferation. In the peak synergy region, a concentration of 2.5 µM for Alisertib was identified as the optimal dose. To determine the optimal Dosage of Donafenib, we assessed cellular viability (Figure 1G) and colony-forming efficacy (Figures 1H,I) across various treatment regimens. Optimal concentrations were established at 10 µM for HCCLM3 cells and 5 µM for Huh7 cells, both showing significant anti-proliferative effects (p < 0.05). After 72 h of combined treatment, we observed a marked reduction in growth rates for both cell lines (Figure 1J, P < 0.001). These findings indicate that Alisertib, at concentrations well below its IC_50_, significantly enhances the cytotoxic effects of Donafenib in HCC cells.

### Combination of Donafenib and Alisertib synergistically induces ferroptosis-driven cell death in HCC

**FIGURE 2:**
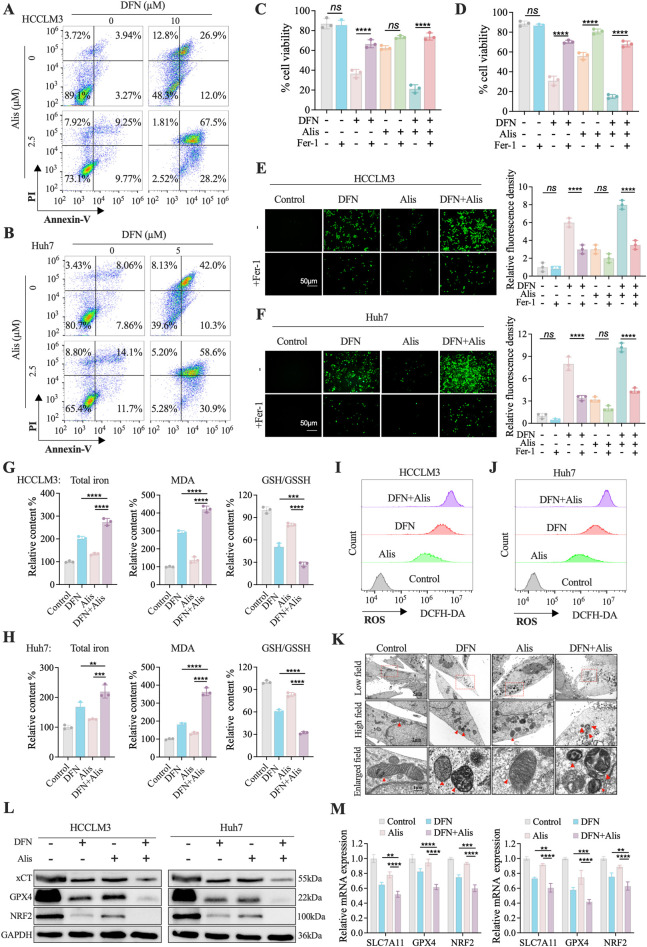
Alisertib and Donafenib induced cell death of HCC cells mainly through ferroptosis. **(A,B)** Quantitative analysis of cell death induced by Donafenib and/or Alisertib by flow cytometry. **(C,D)** Cell viability percentages following treatment with Donafenib and/or Alisertib combined with the ferroptosis inhibitor Fer-1 (10 μM) for 48 h **(E,F)** Relative lipid ROS levels were measured via BODIPY-C11 fluorescence. **(G,H)** Assessment of total iron content, malondialdehyde (MDA), and glutathione (GSH/GSSH) levels. **(I,J)** Relative cytosolic ROS levels were quantified using DCFH-DA fluorescence. **(K)** TEM of HCCLM3 treated with Donafenib and/or Alisertib for 24 h **(L, M)** Protein expressions of xCT, GPX4, and NRF2 were evaluated and quantified through Western blotting and qPCR. (n = 3, *P < 0.05, **P < 0.01, ***P < 0.001, ****P < 0.0001, ns, non-significant; DFN, Donafenib; Alis, Alisertib).

The combination of Donafenib and Alisertib significantly enhances the sensitivity of hepatocellular carcinoma (HCC) cells to Donafenib, resulting in reduced cell proliferation and viability. Flow cytometry analyses demonstrated that co-treatment with Donafenib and Alisertib substantially increased the percentage of Annexin-V + PI + cells in the HCCLM3 and Huh7 cell lines (Figures 2A,B). To elucidate the mechanisms underlying this cell death, we utilized various inhibitors to assess cell viability (Figures 2C,D; Supplementary Figure S1). Inhibitors of autophagy (3-MA), necroptosis (Nec-1), and pyroptosis (VX-765) did not reverse cell death. Conversely, Z-VAD-FMK, an apoptosis inhibitor, slightly mitigated cell death, while Ferrostatin-1 (Fer-1; 10 µM), a ferroptosis inhibitor, significantly restored cell viability compromised by the drug combination (Figures 2C,D). These findings suggest that ferroptosis is the primary mode of cell death.

Furthermore, co-treatment led to a significant accumulation of reactive oxygen species (ROS), which was effectively inhibited by Fer-1 (Figures 2E,F). Notably, levels of total iron and malondialdehyde (MDA) were upregulated, whereas glutathione was downregulated in the combination treatment group (Figures 2G,H). DCFH-DA fluorescence analysis revealed a marked increase in intracellular ROS levels following co-treatment (Figures 2I,J). Transmission electron microscopy (TEM) further illustrated mitochondrial shrinkage, loss of cristae, and increased electron density in membranes, all characteristic of ferroptosis (Figure 2K) (Dixon et al., 2012). Additionally, we observed significant downregulation of key ferroptotic defense molecules, including xCT, GPX4, and NRF2 pathways, indicating a diminished anti-ferroptotic response in the combined treatment group (Figures 2L,M). In brief, our findings establish that the synergistic application of Alisertib and Donafenib effectively induces ferroptosis in HCC cells, underscoring a promising therapeutic strategy for HCC.

### The synergistic effect of Alisertib and Donafenib on ferroptosis in HCC cells mediated by the NF-κB signaling pathway

**FIGURE 3:**
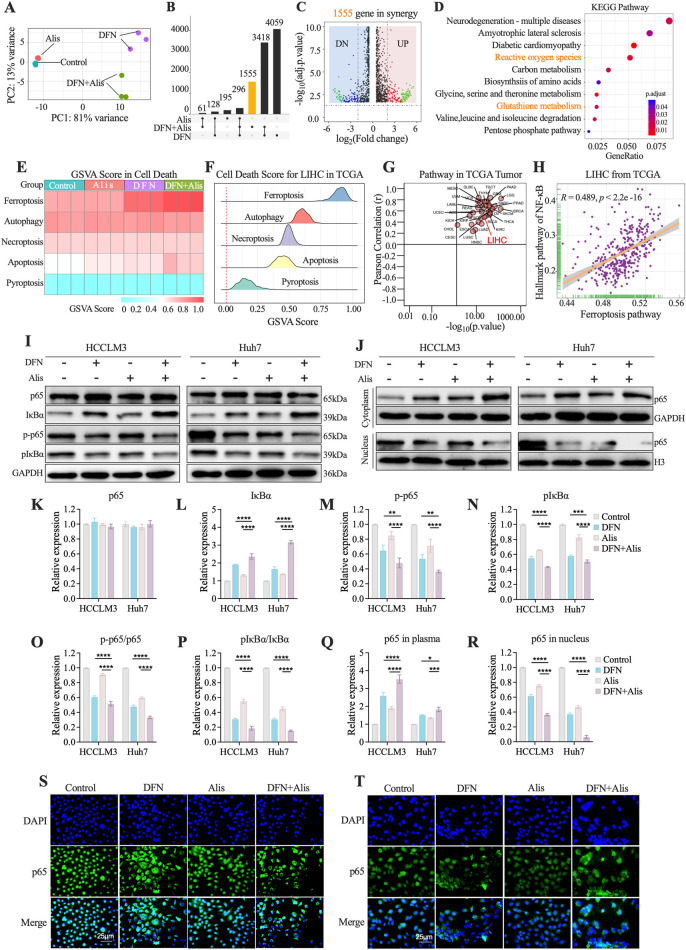
The Synergistic Effect of Alisertib and Donafenib on Ferroptosis in Hepatocellular Carcinoma Cells Mediated by the NF-κB Signaling Pathway. **(A)** PCA analysis of sequencing samples from HCCLM3 cells. **(B)** Upset plot illustrates the DEGs (p < 0.05, |log_2_FC|>1) across treatment groups. **(C)** Volcano plot highlights DEGs significantly in 1555 genes (p < 0.05, |log2FC|>2). **(D)** KEGG enriched pathways in synergy. **(E)** Heatmap for PCD scores across HCCLM3. **(F)** Enrichment scores for PCD in TCGA LIHC samples. **(G)** Correlation between ferroptosis scores and the NF-κB signaling pathway across 33 tumor types in TCGA and **(H)** in LIHC from TCGA. **(I)** NF-κB signaling molecules (p65, IκBα, p-p65, p-IκBα) with quantitative analyses for **(K)** p65, **(L)** IκBα, **(M)** p-p65, **(N)** p-IκBα, and **(O)** ratios of p-p65/p65 and **(P)** p-IkBa/IkBa. **(J)** cytoplasmic and nuclear p65 expression, including quantitative results for **(Q)** cytoplasmic p65 and **(R)** nuclear p65. **(S, T)** p65 nuclear translocation in HCCLM3 and Huh7 cells. (n = 3, *P < 0.05, **P < 0.01, ***P < 0.001, ****P < 0.0001, ns, non-significant; DFN, Donafenib; Alis, Alisertib).

To explore the effects of Donafenib and Alisertib on ferroptosis induction in HCC cells, we performed RNA sequencing to identify differentially expressed genes (DEGs) in HCCLM3 cells treated with each agent alone and in combination. The principal component analysis (PCA) revealed strong intra-group consistency and significant inter-group differentiation (Figure 3A). This strongly suggests that the different treatment regimens considerably altered the global gene expression profiles of the cells.

In the combination group, we identified 1,555 differentially expressed genes (DEGs) excluding genes from individual treatments (p < 0.05, |log_2_FC|>1, Figure 3B; Supplementary Figure S2). The volcano plot highlighted significantly regulated genes in the combination group (|log_2_FC| > 2, p < 0.05, Figure 3C). KEGG enrichment analysis revealed significant enhancement in pathways related to reactive oxygen species metabolism, glutathione metabolism, and the pentose phosphate pathway (Figure 3D), suggesting a close connection to the regulatory mechanisms of ferroptosis (Yao et al., 2021; Liang et al., 2022). Quantitative assessment of ferroptosis-related pathways employed ssGSEA scoring with five established programmed cell death (PCD) gene sets (Figure 3E) (Liu et al., 2022). Ferroptosis scores showed maximal activation in the combination group versus monotherapies (p < 0.05), while apoptosis remained minimally active across treatments. TCGA validation (n = 371 LIHC) confirmed ferroptosis as the dominant PCD mechanism (Figure 3F). Pan-cancer analysis (33 tumor types) revealed NF-κB pathway’s universal correlation with ferroptosis, particularly in HCC (LIHC: r = 0.489, p < 0.05; Figures 3G,H) (Guo et al., 2023). Western blot analyses revealed significantly reduced phosphorylation of both p-p65 (Figures 3I,M) and pIκBα (Figure 3N) in the combination therapy group versus single-agent treatments. Total p65 levels remained stable across groups (Figure 3K), while IκBα protein demonstrated marked elevation in the combination cohort (Figure 3L). Both p-p65/p65 and pIκBα/IκBα ratios exhibited significant reductions (Figures 3O,P). Subcellular localization analysis showed cytoplasmic accumulation alongside nuclear depletion of p65 in combination-treated cells (Figures 3J,Q,R). Immunofluorescence validation confirmed potent suppression of p65 nuclear translocation (Figures 3S,T). Collectively, the Donafenib-Alisertib combination suppresses NF-κB signaling by inhibiting p65 activation and nuclear translocation while stabilizing IκBα, mechanistically driving ferroptosis in HCC.

### *In Vivo* efficacy of Donafenib-Alisertib combination in HCCLM3 xenografts

**FIGURE 4:**
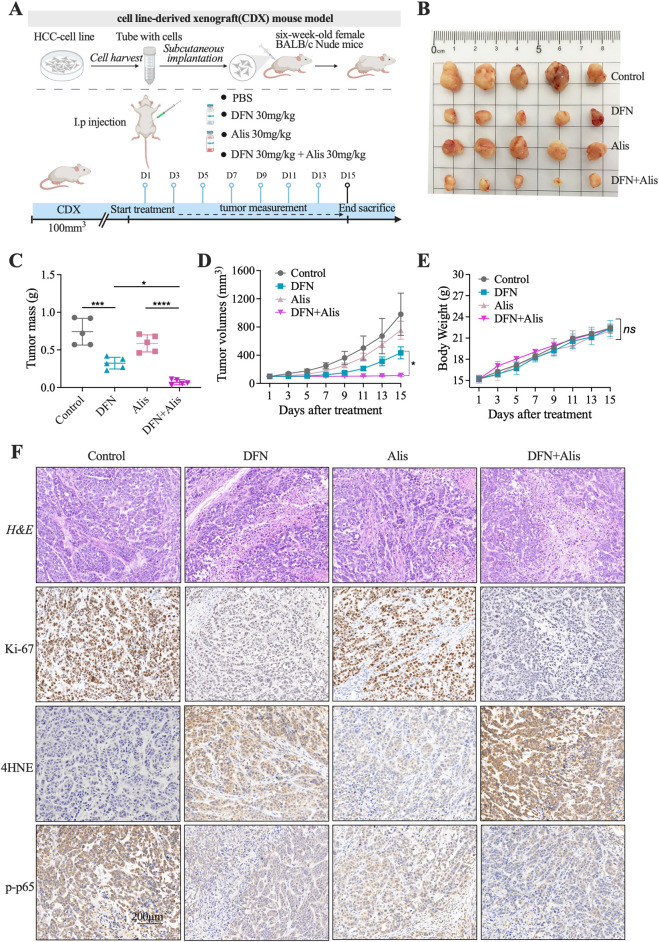
Combined Donafenib and Alisertib therapy suppresses tumor growth. **(A)** Experimental design for xenograft model. **(B)** Representative tumors post-treatment. **(C)** Final tumor weights. **(D)** Tumor growth curves. **(E)** Body weights. **(F)** H&E (necrosis), 4HNE (lipid peroxidation), Ki-67 (proliferation), p-p65 (phospho-p65) staining. (n = 5, *P < 0.05, **P < 0.01, ***P < 0.001, ****P < 0.0001, ns, non-significant; DFN, Donafenib; Alis, Alisertib).

Subcutaneous HCCLM3 xenograft models were established to assess the antitumor effects of Donafenib (30 mg/kg) combined with Alisertib (30 mg/kg). Tumor-bearing mice (baseline volume: 100 mm^3^) received monotherapy or combination therapy (Figure 4A). The combination group demonstrated significantly reduced tumor volume compared to monotherapy groups (Figures 4B,D) and suppressed tumor growth relative to controls (Figure 4C). Body weights remained stable across all groups (p > 0.05, Figure 4E). Tumors from the combination group exhibited elevated 4HNE immunostaining (Figure 4F), indicating enhanced lipid peroxidation. H&E staining confirmed increased tumor necrosis, while Ki-67 staining revealed reduced proliferation. Consistent with *in vitro* findings, p-p65 immunostaining showed decreased NF-κB activation in the combination group.

### PMA restores NF-κB signaling to reverse ferroptosis induced by Donafenib and Alisertib

**FIGURE 5:**
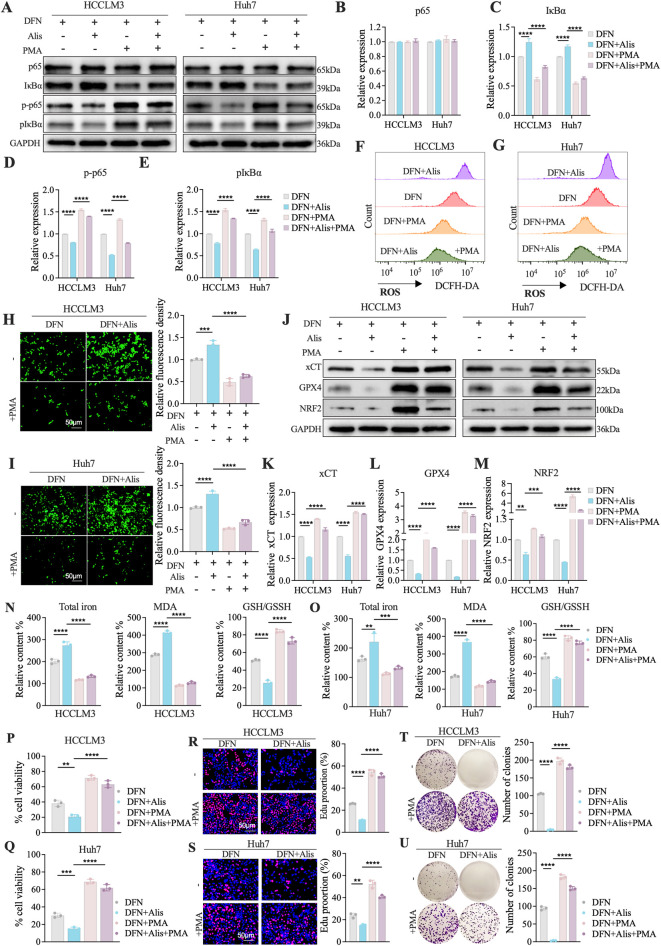
PMA restores NF-κB signaling to reverse ferroptosis induced by Donafenib and Alisertib. **(A–E)** Representative Western blot analysis of NF-κB signaling activation. **(F,G)** Measurement of relative cytosolic reactive oxygen species (ROS) levels. **(H,I)** Assessment of lipid ROS levels. **(J)** Western blot analysis of ferroptosis-related proteins. **(K–M)** qPCR analysis of xCT, GPX4, and NRF2 expression levels. **(N,O)** Relative levels of total iron content, malondialdehyde (MDA), and GSH/GSSG ratio. **(P,Q)** Evaluation of cell viability. **(R,S)** Assessment of proliferative capacity. **(T,U)** Analysis of colony formation ability. (n = 3, *P < 0.05, **P < 0.01, ***P < 0.001, ****P < 0.0001, ns, non-significant; DFN, Donafenib; Alis, Alisertib).

Co-treatment with the NF-κB activator PMA (100 nM, 24 h) rescued NF-κB pathway inhibition caused by Donafenib and Alisertib in HCCLM3 and Huh7 cells. Western blot analysis showed PMA increased phosphorylation of p65 and IκBα while reducing total IκBα levels (Figures 5A–E), confirming restoration of NF-κB activity that reversed drug-induced blockade of p65 nuclear translocation and IκBα degradation.

The ferroptosis phenotype was systematically reversed by PMA: Both cytosolic ROS (DCFH-DA assay) and lipid ROS (BODIPY-C11) levels decreased significantly (Figures 5F–I), with concurrent upregulation of ferroptosis defense proteins xCT, GPX4, and NRF2 (Figures 5J–M). PMA also reduced total iron content and malondialdehyde (MDA) levels while increasing the GSH/GSSG ratio (Figures 5N,O). Functionally, PMA restored cell viability (Figures 5P,Q), increased EdU + proliferating cells (Figures 5R,S), and enhanced colony formation capacity (Figures 5T,U). Therefore, Pharmacological NF-κB reactivation via PMA counteracted the pro-ferroptotic synergy effects of Donafenib and Alisertib, demonstrating that synergistic ferroptosis induction by these agents requires NF-κB pathway inhibition.

### Donafenib and Alisertib synergistically block NRF2 transcription through NF-κb/p65

**FIGURE 6:**
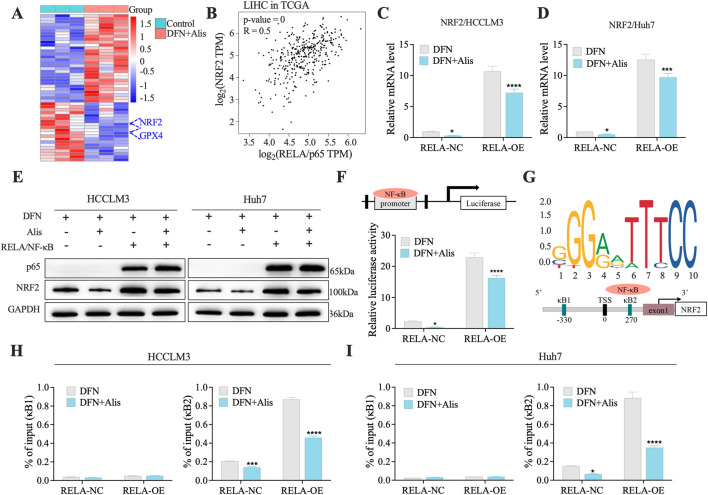
Donafenib and Alisertib synergistically Block NRF2 Transcription through NF-κB/p65 **(A)**. Heatmap of the top 50 DEGs in synergy. **(B)** Correlation analysis of NRF2 and RELA from TCGA dataset. **(C,D)** qPCR analysis of NRF2 in HCC cell lines. **(E)** Representative Western blot of NRF2 expression in RELA-overexpressing cells treated with Donafenib alone or in combination with Alisertib. **(F)** Relative luciferase activity in 293T cells treated with Donafenib alone or in combination with Alisertib. **(G)** Schematic representation of the Nrf2 promoter constructs. **(H,I)** ChIP-qPCR results illustrating the enrichment of the NRF2 promoter at the kB1 and kB2 sites. (n = 3, *P < 0.05, **P < 0.01, ***P < 0.001, ****P < 0.0001, ns, non-significant; DFN, Donafenib; Alis, Alisertib).

To better understand the mechanisms behind the synergistic effects of donafenib and alisertib on HCC, we conducted a detailed analysis of the top 50 differentially expressed genes (DEGs) identified from RNA sequencing data, focusing specifically on ferroptosis-related markers. Notably, we found that NRF2 expression was significantly downregulated after combination therapy, which is closely linked to the induction of ferroptosis (Figure 6A). Additionally, GPX4, a key enzyme involved in NRF2-regulated glutathione (GSH) synthesis, showed altered expression, indicating a reduced capacity for cellular antioxidant defense (Anandhan et al., 2020).

Given that NRF2 is transcriptionally activated by RELA/NF-κB (Rushworth et al., 2012; Wang et al., 2021), we performed a correlation analysis using data from the TCGA-HCC dataset. This analysis revealed a significant positive correlation between the mRNA levels of NRF2 and RELA (NF-κB/p65) (Figure 6B), suggesting an interplay between the NRF2 and NF-κB signaling pathways in the context of ferroptosis. To further validate this interaction, we overexpressed RELA in HCC cell lines and evaluated its impact on ferroptosis-related gene expression through qPCR and Western blot analyses. Notably, the upregulation of NRF2 correlated with RELA induction, an effect that was significantly reduced in the presence of the drug combination (Figures 6C–E). Subsequent mechanistic studies using dual-luciferase reporter assays confirmed that p65 directly regulates NRF2 promoter activity (Figure 6F). Additionally, chromatin immunoprecipitation (ChIP) assays (Figure 6G) showed that combination treatment significantly decreased RELA/NF-κB occupancy at the NRF2 promoter in HCC cells, particularly at the kB2 site (Figures 6H,I), providing direct evidence of transcriptional regulation. Collectively, these findings underscore the critical role of the NF-κB/NRF2 axis in regulating ferroptosis, highlighting its potential as a promising therapeutic target in HCC.

### *In Vivo* rescue of antitumor efficacy in combination therapy mediated by NF-κB pathway activation

**FIGURE 7:**
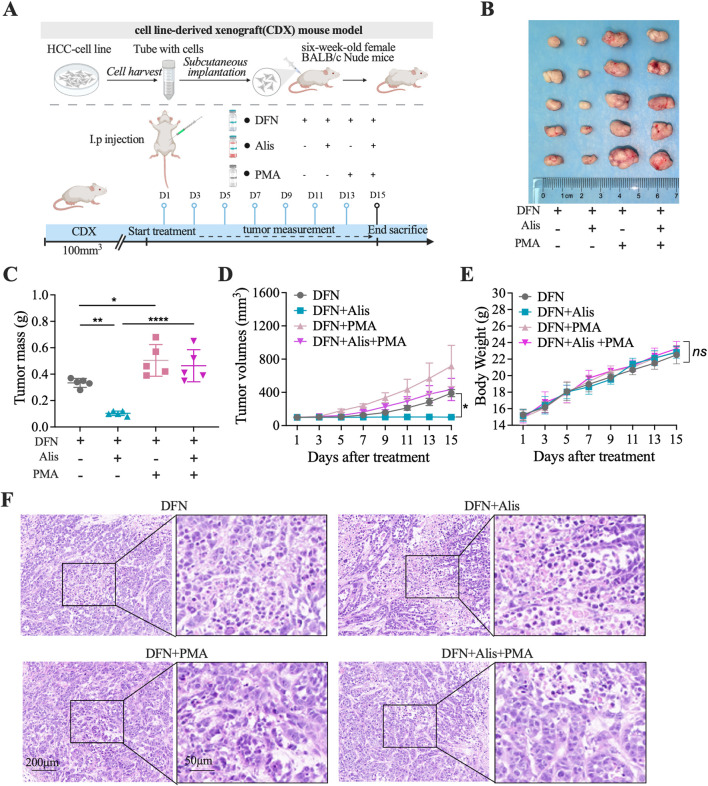
The antitumor effects of Alisertib in synergy with Donafenib are reinstated *in vivo* via the activation of the NF-κB signaling pathway. **(A)** Treatment protocol for subcutaneously implanted tumor-bearing mice. **(B)** Images of tumors excised from different treatment groups. **(C)** Tumor weight comparison, **(D)** tumor volume, and **(E)** body weight across mouse cohorts. **(F)** Histopathological evaluation via HE staining. (n = 5, *P < 0.05, **P < 0.01, ***P < 0.001, ****P < 0.0001, ns, non-significant; DFN, Donafenib; Alis, Alisertib).

To further investigate the role of the NF-κB signaling cascade in mediating the synergistic effects of this combination treatment, we conducted rescue experiments using PMA (300 μg/kg) in mice with subcutaneously implanted tumors. These mice were treated either with Donafenib monotherapy or with Donafenib in combination with Alisertib, as shown in Figure 7A. The results demonstrated that the antitumor efficacy observed in both the Donafenib and combination treatment groups was significantly reversed following PMA exposure (Figure 7B). Specifically, PMA administration led to a substantial increase in tumor volume in both treatment regimens, highlighting a marked reduction in the synergistic antitumor effects (Figures 7C,D).

Body weight measurements across the groups showed no statistically significant differences, indicating an acceptable tolerance profile (Figure 7E). Furthermore, histopathological analysis revealed that PMA treatment resulted in a significant decrease in the necrotic rate within the tumor tissue (Figure 7F). Collectively, these *in vivo* rescue experiments suggest that the efficacy of the combination therapy heavily relies on the inhibition of NF-κB signaling pathway activity to facilitate ferroptosis. The sensitization of HCC cells to Donafenib by Alisertib may pave the way for novel therapeutic strategies in the treatment of HCC.

### *In Vivo* toxicity analysis

**FIGURE 8:**
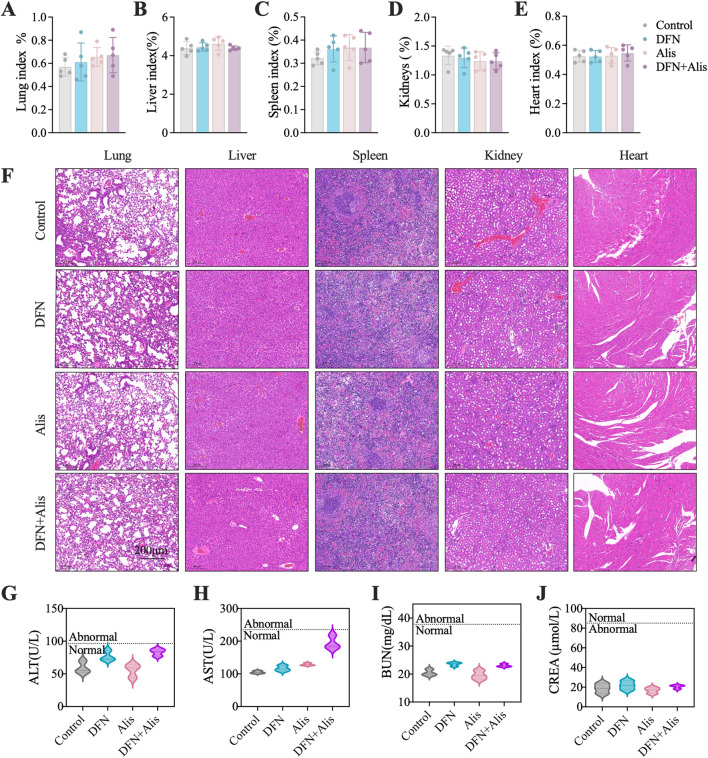
Evaluation of *in vivo* toxicity profiles. Organ index, including **(A)** lung, **(B)** liver, **(C)** spleen, **(D)** kidney, and **(E)** heart, were meticulously evaluated at the end of the *in vivo* experiment. **(F)** HE staining was performed to examine the histopathological characteristics of the organs in nude mice. Assessments of systemic hepatic and renal function included measurements of **(G)** alanine aminotransferase (ALT), **(H)** aspartate aminotransferase (AST), **(I)** creatinine (Cr), and **(J)** blood urea nitrogen (BUN). (n = 5, *P < 0.05, **P < 0.01, ***P < 0.001, ****P < 0.0001, ns, non-significant; DFN, Donafenib; Alis, Alisertib).

The systemic toxicity of the different treatment groups was evaluated through monitoring body weight changes and assessing the histopathological integrity of major organs in nude mice. Over time, all treatment groups exhibited an increase in body weight, attributed to tumor enlargement. Importantly, our findings indicated that neither Donafenib administered alone nor the combination of Donafenib with Alisertib resulted in significant weight loss, and intergroup differences were not statistically significant. This suggests that the dosages of Donafenib and Alisertib utilized in our study are within a safe range. Furthermore, we conducted thorough dissection, weighing, and H&E staining of the principal organs of the mice to evaluate any potential adverse effects. As depicted in Figures 8A–E, we calculated organ indices using the formula: Organ Index (%) = [(Organ Weight)/(Body Weight of Mouse)] × 100%. Optical microscopy was utilized to examine and capture images of the organ sections, as illustrated in Figure 8F. The findings revealed normal structural morphology of the major organs, devoid of any discernible pathological changes, indicating that the administered drugs did not elicit systemic toxicity. Additionally, we performed biochemical analyses on blood samples collected from the orbital sinus of the mice, as illustrated in Figures 8G–J. The serum levels of ALT, AST, CREA, and BUN remained within normal reference ranges, indicating that hepatic and renal functions were unaffected by the treatments. Collectively, these findings substantiate the robust safety profile of our therapeutic regimen.

## Discussion

HCC is recognized as one of the most widespread cancers globally. There have been major advancements in targeted treatments for HCC, starting with the launch of sorafenib, the inaugural small-molecule targeted anti-cancer drug (Llovet et al., 2008). Following this breakthrough, additional therapies such as Lenvatinib (Kudo et al., 2018), regorafenib (Bruix et al., 2017), and Donafenib (Qin et al., 2021) have come into play. However, despite these developments, Lenvatinib and regorafenib have not demonstrated significant enhancements in patient survival rates. In contrast, Donafenib appears to provide a modest benefit compared to sorafenib, with an increase in overall survival of around 1.8 months. Nevertheless, targeted therapies for HCC encounter considerable hurdles owing to prevalent mutations, including those in the TERT promoter and TP53, which complicate therapeutic targeting (Zheng C. et al., 2023; Wang et al., 2018). This scenario emphasizes the critical need for novel treatment approaches.

**FIGURE 9:**
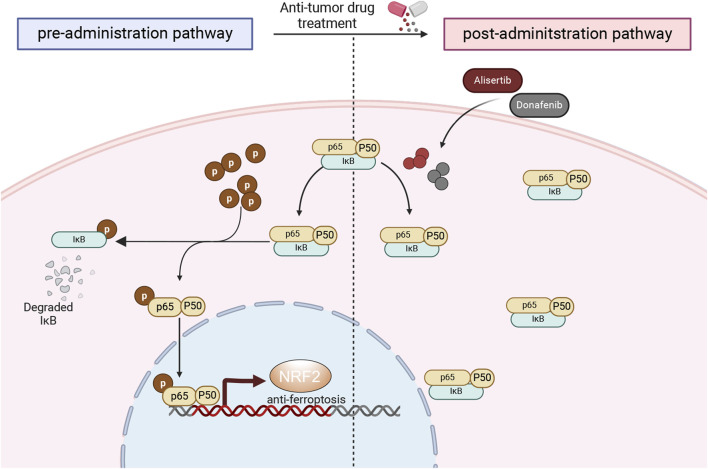
Mechanistic overview of cytotoxicity induced by the synergistic interaction of donafenib and alisertib.

In our investigation, we assessed the impact of the Aurora-A kinase inhibitor Alisertib alongside Donafenib on HCC, focusing on the mechanisms that facilitate their synergistic induction of ferroptosis in the HCCLM3 and Huh7 cells, as well as in subcutaneous xenograft models. Additionally, a comprehensive analysis of transcriptome sequencing data revealed that the combination therapy significantly reduces NF-κB signaling pathway activity, which is pivotal in tumor growth and cellular survival. The combination of Alisertib and Donafenib specifically inhibits p65 nuclear translocation and IκBα degradation, preventing NRF2 transcription associated with ferroptosis defense and enhancing ferroptosis-driven cytotoxicity (Figure 9).

Since the introduction of the ferroptosis concept, therapeutic strategies aimed at harnessing this process to effectively eliminate tumors have been increasingly proposed by researchers (Ru et al., 2024; Lei et al., 2022). These approaches consist of reversing radioresistance, improving the sensitivity of molecular-targeted therapies, and enhancing responses to immunotherapy (Roh et al., 2016; Lei et al., 2020; Louandre et al., 2013; Zhao L. et al., 2022). Preclinical research has demonstrated that combining Donafenib with GSK-J4, a histone demethylase KDM6A/B inhibitor, produces a synergistic lethality effect in HCC across various models, including cellular systems, organoids, and subcutaneous xenografts derived from patients and mutant mouse models (Zheng C. et al., 2023). This synergistic effect occurs due to the disruption of intracellular Fe^2+^ levels, which leads to ferroptosis. Aligning with these results, our investigation noted a significant reduction in both short-term cell viability and long-term clonogenic capability after treating with a combination of Alisertib and Donafenib at safe doses. Moreover, the cytotoxicity from this combination was effectively prevented by the ferroptosis inhibitor Fer-1, supporting the observed accumulation of reactive oxygen species (ROS), lipid peroxides, Fe^2+^ ions, and a drop in GSH/GSSH ratios, which are characteristic indicators of ferroptosis (Tang and Kroemer, 2020). This underscores the potential of ferroptosis as a novel therapeutic strategy for treating HCC.

Recent research has placed significant emphasis on the potential applications of Aurora-A kinase inhibitors in treating a variety of malignancies. Notably, results from phase III clinical trials in lymphoma have illustrated efficacy of Alisertib, thus paving the way for further clinical translation and exploration (Zhou et al., 2024; O'Connor et al., 2019). Building on the established understanding of how aberrantly expressed Aurora-A contributes to oncogenesis through its involvement in crucial mitotic processes—particularly centrosome separation, maturation, and spindle assembly—our previous investigations have shown that reducing Aurora-A expression can lead to a substantial inhibition of HCC cell proliferation. This downregulation not only induces G2/M cell cycle arrest but also significantly promotes apoptotic activities within these cells (Gao et al., 2008; Fu et al., 2007). In addition, we have conducted comprehensive studies focused on understanding the factors contributing to resistance against targeted therapies in HCC. This research delved into genetic elements at various levels, including cDNA, miRNA, and lncRNA. Our findings revealed that certain pathways, notably the PU.1/microRNA-142-3p/ATG5/ATG16L1 and the FOXM1/LINC-ROR feedback loops, are pivotal regulators influencing HCC cell sensitivity to sorafenib, specifically through their roles in modulating autophagy and cellular proliferation (Zhi et al., 2019; Zhang et al., 2018a). Moreover, our analysis concerning the role of Aurora-A expression on therapeutic responses in HCC cells uncovered that Aurora-A plays a significant role in mediating radioresistance and chemoresistance via the regulation of the NF-κB signaling pathway (Shen et al., 2019; Zhang et al., 2014). Expanding on this foundational knowledge, our current study is the first to provide compelling evidence that combining Aurora-A inhibitors with Donafenib significantly inhibits the activation of the NF-κB signaling pathway. Consistent with previous studies, NF-κB is essential for enhancing TNFα-induced Nrf2 protein levels and the expression of its target genes (Wardyn et al., 2015). Our research further demonstrates that NF-κB, a well-characterized transcription factor, mediates the transcriptional upregulation of Nrf2 related to ferroptosis effects. The significant alteration in NRF2 reporter gene activity further corroborates the inhibitory effect of combination therapy on Nrf2 transcriptional activity. Within the proximal promoter region of Nrf2, two κB binding sites have been identified, with κB2 located at +270 upstream of the Nrf2 transcription start site, capable of binding NF-κB/p65 (Rushworth et al., 2012; Wang et al., 2021). Our ChIP assays further confirm that the combination therapy synergistically inhibits the phosphorylation and nuclear translocation of NF-κB molecules. This inhibition leads to the downregulation of Nrf2 expression, subsequently promoting the upregulation of ferroptosis-induced cytotoxic effects.

Additionally, when assessing systemic toxicity associated with this combination therapy, we observed a commendable safety profile, with no noticeable behavioral abnormalities in mice throughout the treatment period. Given the established dosing regimens and pharmacokinetic profiles of both agents, further studies are warranted to evaluate patient selection criteria, such as specific molecular markers associated with the NF-κB/NRF2 pathway, which could better predict treatment response and optimize clinical outcomes.

Several limitations of this study warrant consideration. First, while our *in vivo* and *in vitro* assays demonstrate significant efficacy, established models may not fully capture the tumor heterogeneity, particularly the immune context observed in patients, which limits our understanding of treatment-related immune responses. Additionally, the use of PMA as a broad protein kinase C (PKC) activator raises concerns about specificity, as it may inadvertently activate pathways unrelated to NF-κB. Importantly, we recognize that the regulation of ferroptosis involves mechanisms beyond the NF-κB/NRF2-mediated defense system, highlighting the complexity of this regulatory network and the potential presence of feedback loops. Future studies should focus on specific NF-κB activators or inhibitors, explore interactions among various signaling pathways, and utilize immunocompetent mouse models and patient-derived organoid systems to better mimic the tumor microenvironment and immune responses in patients.

## Conclusion

In summary, HCC remains a formidable challenge in cancer treatment, necessitating innovative therapeutic strategies. Our study highlights the synergistic efficacy of the Aurora-A kinase inhibitor, Alisertib, in combination with Donafenib, demonstrating their ability to promote ferroptosis through the downregulation of NF-κB signaling. This dual therapeutic approach not only enhances cytotoxicity against HCC cells but also addresses critical resistance mechanisms that contribute to treatment failure. The favorable safety profile observed in our preclinical models further supports the potential for clinical application of this combinatorial strategy. Ultimately, our findings advocate for the development of novel treatment paradigms that integrate molecular-targeted therapies, which could significantly improve patient outcomes and survival rates in HCC. Further research is warranted to translate these promising results into clinical practice.

## Data Availability

The datasets presented in this study can be found in online repositories. The names of the repository/repositories and accession number(s) can be found in the article/Supplementary Material.
