# Correction: Cooperative targeting of NF-κB enhances ferroptosis-driven HCC therapy with Alisertib and Donafenib

**DOI:** 10.3389/fcell.2025.1685448

**Published:** 2025-09-10

**Authors:** Qiong Zhou, Rui Wang

**Affiliations:** Laboratory of Medical Oncology, Jinling Hospital, Affiliated Hospital of Medical School, Nanjing University, Nanjing, Jiangsu, China

**Keywords:** hepatocellular carcinoma, Donafenib, Alisertib, ferroptosis, NF-κB signaling pathway

There was a mistake in Figure 3I as published. During the proofing process, the image intended to represent the p65 WB bands from Huh7 cells was mistakenly duplicated, which resulted in the appearance of incorrect images in Figure 3I. We have since conducted a thorough review of all original bands and have confirmed that the original images were accurate and did not contain any duplication. This error was unintentional and occurred during the image compilation. Importantly, this correction does not affect the conclusions drawn from our experiments. The corrected Figure 3 appears below.

**FIGURE 3:**
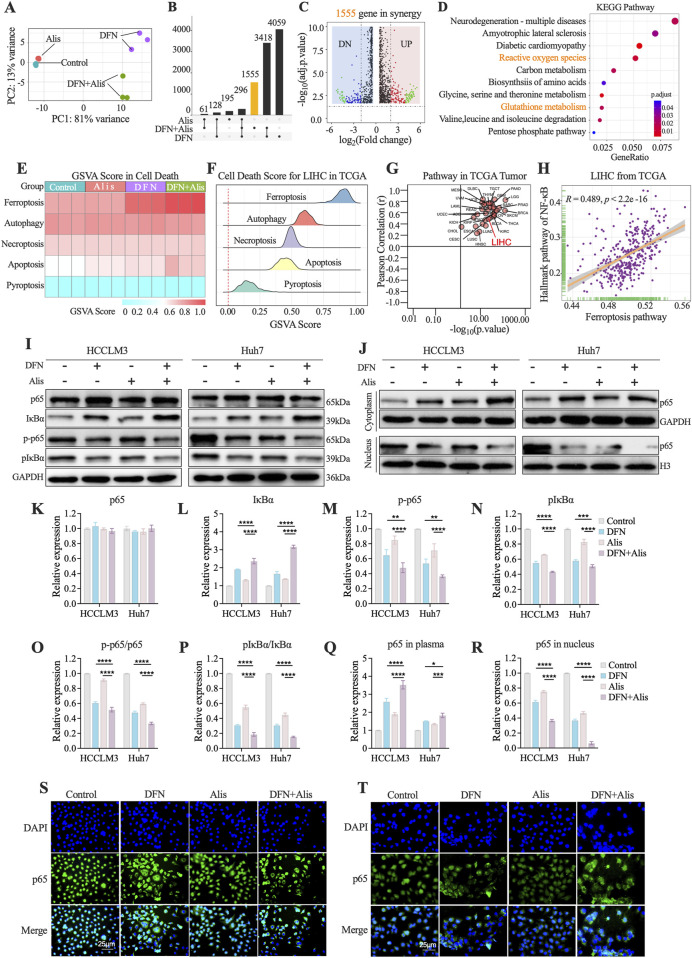
The Synergistic Effect of Alisertib and Donafenib on Ferroptosis in Hepatocellular Carcinoma Cells Mediated by the NF-κB Signaling Pathway. **(A)** PCA analysis of sequencing samples from HCCLM3 cells. **(B)** Upset plot illustrates the DEGs (p < 0.05, |log_2_FC|>1) across treatment groups. **(C)** Volcano plot highlights DEGs significantly in 1555 genes (p < 0.05, |log2FC|>2). **(D)** KEGG enriched pathways in synergy. **(E)** Heatmap for PCD scores across HCCLM3. **(F)** Enrichment scores for PCD in TCGA LIHC samples. **(G)** Correlation between ferroptosis scores and the NF-κB signaling pathway across 33 tumor types in TCGA and **(H)** in LIHC from TCGA. **(I)** NF-κB signaling molecules (p65, IκBα, p-p65, p-IκBα) with quantitative analyses for **(K)** p65, **(L)** IκBα, **(M)** p-p65, **(N)** p-IκBα, and **(O)** ratios of p-p65/p65 and **(P)** p-IkBa/IkBa. **(J)** cytoplasmic and nuclear p65 expression, including quantitative results for **(Q)** cytoplasmic p65 and **(R)** nuclear p65. **(S, T)** p65 nuclear translocation in HCCLM3 and Huh7 cells. (n = 3, *P < 0.05, **P < 0.01, ***P < 0.001, ****P < 0.0001, ns, non-significant; DFN, Donafenib; Alis, Alisertib).

The original version of this article has been updated.

