# Supplementary material for: Cooperative targeting of NF-κB enhances ferroptosis-driven HCC therapy with Alisertib and Donafenib
Source: Front Cell Dev Biol. 2025 Aug 13;13:1637767. doi: 10.3389/fcell.2025.1637767 (PMC12380753; doi:10.3389/fcell.2025.1637767)
Supplement: Supplementary file 1 [file DataSheet1.docx]

**Supplemental Materials**

**Table S1** Primary antibodies involved in our study

| Antibodies | Company | Catalog number |
| --- | --- | --- |
| xCT/SLC7A11 | CST | #98051 |
| GPX4 | CST | #52455 |
| NRF2 | CST | #20733 |
| p65 | CST | #8242 |
| p-P65 | CST | #3033 |
| IκBα | CST | #9242 |
| p- IκBα | CST | #2859 |
| 4HNE | Abcam | ab48506 |
| GAPDH | proteintech | 10494-1 |
| H3 | proteintech | 81984-2 |

**Table S2** Primers in our study

| primer | Sequence ((5'to3')) |
| --- | --- |
| h-GAPDH-F | GTCTCCTCTGACTTCAACAGCG |
| h-GAPDH-R | ACCACCCTGTTGCTGTAGCCAA |
| h-SLC7A11-F | TCCTGCTTTGGCTCCATGAACG |
| h-SLC7A11-R | AGAGGAGTGTGCTTGCGGACAT |
| h-NRF2-F | CACATCCAGTCAGAAACCAGTGG |
| h-NRF2-R | GGAATGTCTGCGCCAAAAGCTG |
| h-GPX4-F | GAGGCAAGACCGAAGTAAACTAC |
| h-GPX4-R | CCGAACTGGTTACACGGGAA |

| 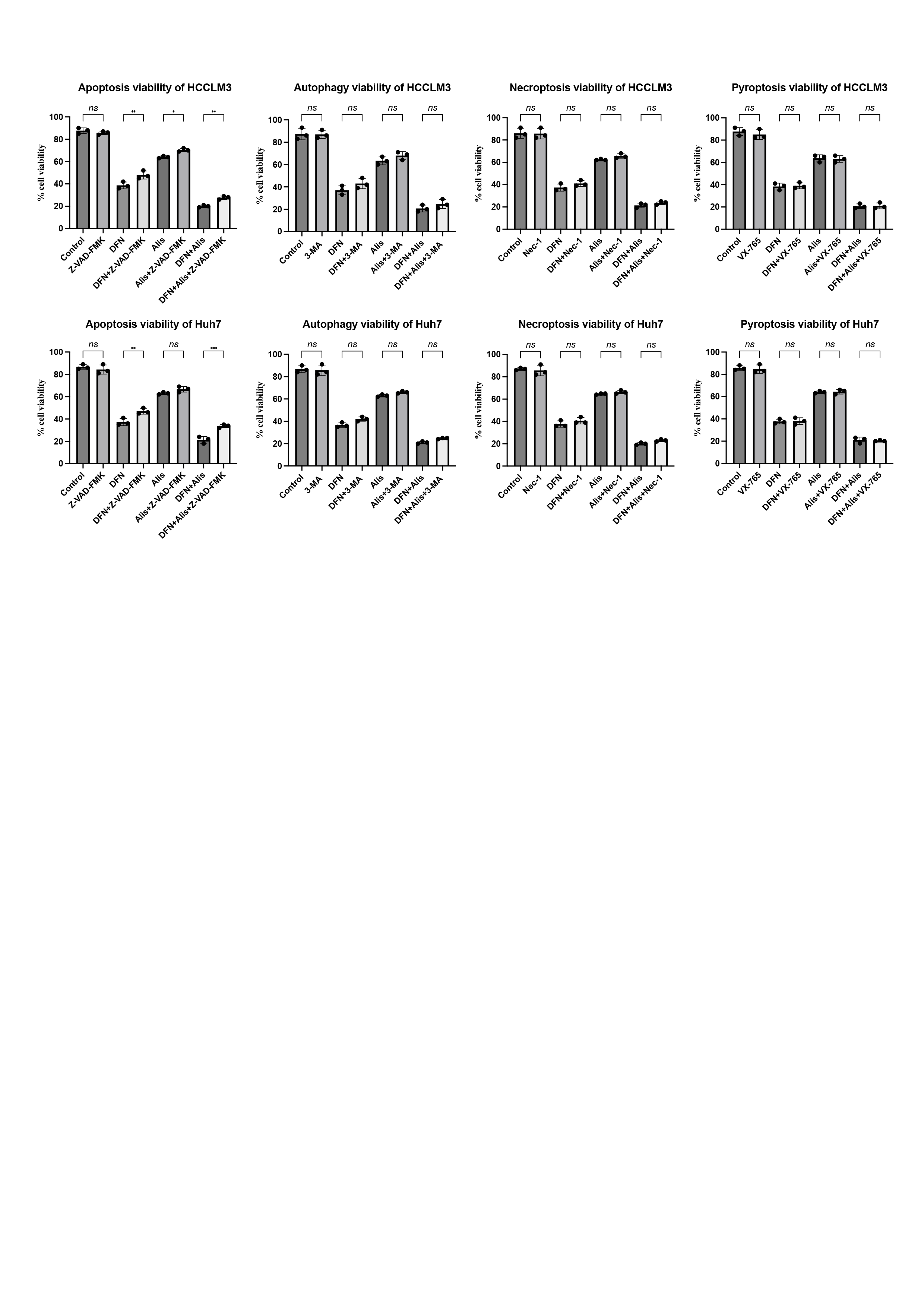 |
| --- |
| **Figure S1 Various inhibitors to assess cell viability.** Notably, autophagy (3-MA), necroptosis (Nec-1), and pyroptosis (VX-765) inhibitors did not reverse the induced cell death, meanwhile, Z-VAD-FMK (apoptosis inhibitor) slightly mitigated cell death. ** P < 0.05, ** P < 0.01, *** P < 0.001, **** P < 0.0001.* *ns*, nonsignificant. |

| 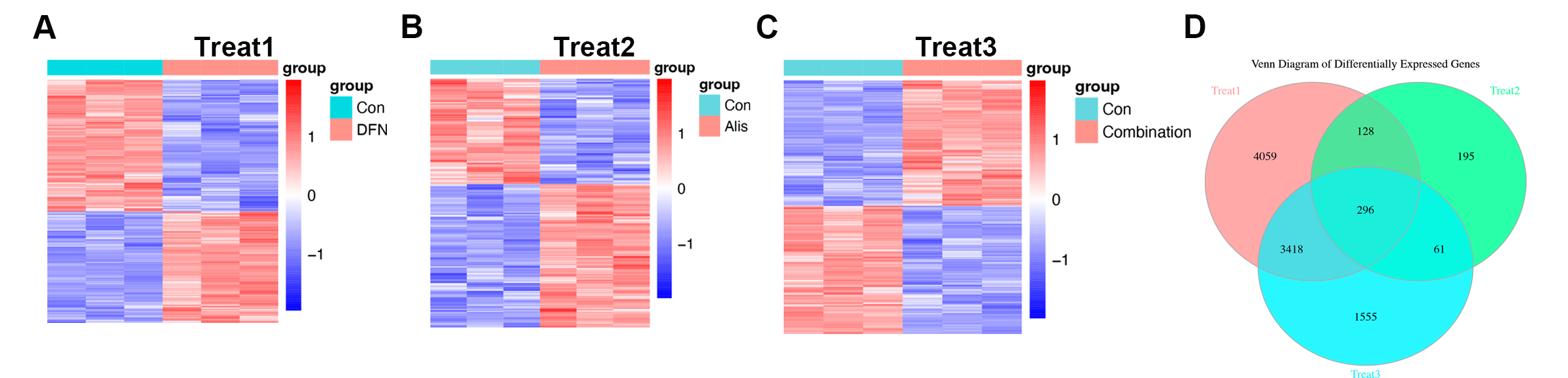 |
| --- |
| **Figure S2: Differential analysis heatmap and Venn diagram comparing treatment groups relative to control.** (A)DFN vs. control; (B) Alis vs. control; (C) DFN combined with Alis vs. control. (D) Venn diagram based on differentially expressed genes. |
